# Evaluating the Risk of Coagulopathy in Cephalosporins with Different Side Chains: A Propensity Score–Weighted Study

**DOI:** 10.3390/medicina62030519

**Published:** 2026-03-11

**Authors:** Chien-Hsiang Tai, Fu-Wen Liang, Chen-Hsiang Lee

**Affiliations:** 1 Division of Infectious Diseases, Department of Internal Medicine, Kaohsiung Municipal Ta-Tung Hospital, Kaohsiung 801, Taiwan; idchtai@gmail.com; 2 Division of Infectious Diseases, Department of Internal Medicine, Kaohsiung Chang Gung Memorial Hospital, Kaohsiung 833, Taiwan; 3 Center for General Education, Cheng Shiu University, Kaohsiung 833, Taiwan; 4 Department of Public Health, College of Health Sciences, Kaohsiung Medical University, Kaohsiung 807, Taiwan; 5 Department of Medical Research, Kaohsiung Medical University Hospital, Kaohsiung Medical University, Kaohsiung 807, Taiwan; 6 Center for Big Data Research, Kaohsiung Medical University, Kaohsiung 807, Taiwan; 7 Division of Infectious Diseases, Department of Internal Medicine, Chiayi Chang Gung Memorial Hospital, Chiayi 613, Taiwan; 8 College of Medicine, Chang Gung University, Taoyuan 333, Taiwan

**Keywords:** coagulopathy, cefazolin, cefoperazone, flomoxef, hypoprothrombinemia

## Abstract

*Background and Objectives*: Cephalosporins containing *N*-hydroxyethyltetrazolethiol (HTT) or *N*-methylthiotetrazole (NMTT) side chains, such as flomoxef and cefoperazone, have been linked to coagulation abnormalities. Cefazolin, which contains an *N*-methylthiadiazolethiol (MTD) side chain, may also interfere with vitamin K metabolism. However, comparative clinical evidence remains limited. This study evaluated the associations between selected cephalosporins and coagulopathy risk. *Materials and Methods*: We conducted a retrospective cohort study using a comprehensive clinical database. Patients receiving cefazolin, flomoxef, or cefoperazone–sulbactam were compared with those receiving reference antibiotics. Coagulopathy was defined as either a ≥25% increase in prothrombin time (PT) from baseline or a PT exceeding the upper limit of normal by more than 3 s within three days before or after antibiotic cessation. Inverse probability of treatment weighting based on propensity scores was applied. Weighted logistic regression was used to estimate odds ratios (ORs) and 95% confidence intervals (CIs). *Results*: After weighting, no significant association with coagulopathy was observed for cefazolin (OR, 1.05; 95% CI, 0.86–1.29), flomoxef (OR, 1.00; 95% CI, 0.77–1.29), or cefoperazone–sulbactam (OR, 0.88; 95% CI, 0.67–1.15). Although international normalized ratio >1.2 was more frequent with cefoperazone–sulbactam, the risk of bleeding events showed a marginal increase compared with the reference group (OR, 1.06; 95% CI, 1.00–1.11). *Conclusions*: Cefoperazone–sulbactam was associated with more frequent laboratory INR elevation and a borderline increase in bleeding risk. Given the observational design, these findings should be interpreted cautiously, and close clinical monitoring may be considered when prescribing cefoperazone–sulbactam.

## 1. Introduction

Bacterial infections contribute substantially to global mortality each year [1]. Notably, third-generation cephalosporin–resistant *Klebsiella pneumoniae*, third-generation cephalosporin–resistant *Escherichia coli*, and carbapenem-resistant *Acinetobacter baumannii* are each estimated to cause 50,000–100,000 deaths annually worldwide [2]. Flomoxef, an *N*-hydroxyethyltetrazolethiol (HTT)-side-chain-containing cephalosporin, has maintained its effectiveness against certain ceftriaxone-resistant pathogens [3]. Moreover, flomoxef has been demonstrated to be comparably efficacious as ertapenem for cefotaxime-resistant Enterobacteriaceae bacteremia [4]. As a result, flomoxef has emerged as a carbapenem-sparing option for infections caused by extended-spectrum ß-lactamase (ESBL)-producing *K. pneumoniae* and *E. coli* [5,6]. Cefoperazone-sulbactam, a combination of ß-lactam and ß-lactamase inhibitors, has shown clinical effectiveness in bloodstream infections and severe pneumonia, including hospital-acquired and ventilator-associated pneumonia [7,8,9,10]. This agent is particularly potent against multidrug-resistant organisms, including ESBL-producing Enterobacteriaceae and carbapenem-resistant *Acinetobacter baumannii*, attributable to sulbactam’s intrinsic activity against *A. baumannii* [11,12,13].

However, the increasing clinical use of flomoxef and cefoperazone–sulbactam raises concerns regarding potential adverse effects. Cephalosporins containing the *N*-methylthiotetrazole (NMTT) side chain have been associated with coagulopathy in experimental and clinical studies [14,15,16]. Vitamin K–dependent coagulation factor activation requires γ-carboxylation of glutamate residues, a process dependent on the vitamin K cycle. Inhibition of vitamin K 2,3-epoxide reductase by the NMTT side chain disrupts this cycle, leading to impaired synthesis of functional vitamin K–dependent coagulation factors [17,18]. Cefoperazone, an NMTT-containing cephalosporin, has been widely associated with hypoprothrombinemia and prolonged prothrombin time (PT) [19]. Similarly, flomoxef, with its HTT side chain, has been linked to hypoprothrombinemia in experimental models, although its inhibitory effect on vitamin K 2,3-epoxide reductase appears weaker than that of NMTT-containing agents [20,21]. Cefazolin, which contains an *N*-methylthiadiazolethiol (MTD) side chain, has likewise been reported to interfere with the vitamin K cycle and may increase the risk of coagulopathy [16,22]. Despite these observations, the clinical relevance of coagulation abnormalities and hemorrhagic events associated with MTD-, HTT-, or NMTT-containing cephalosporins remains unclear, and risk factors for individual agents have not been well defined [23]. Evidence for flomoxef is largely limited to animal studies, and data for cefazolin are primarily derived from case reports [21,24,25]. In Taiwan, cefazolin, flomoxef, and cefoperazone–sulbactam are commonly used in clinical practice. Given the limited and heterogeneous evidence regarding their coagulation-related risks, this study aimed to evaluate the associations between these agents and the risks of coagulopathy and bleeding.

## 2. Materials and Methods

### 2.1. Data Source

The retrospective cohort study was conducted using the Chang Gung Research Database (CGRD), an electronic medical record database derived from the Chang Gung Memorial Hospital healthcare system. The CGRD covers approximately 10% of medical care services reimbursed under the Taiwan National Health Insurance program and incorporates healthcare data from five local hospitals and two medical centers in Taiwan [26]. The database provides comprehensive information, including patient diagnoses, physician orders, prescriptions, and results of laboratory tests, imaging studies, and other examinations across outpatient, emergency, and inpatient settings.

### 2.2. Study Cohort

Adult inpatients recorded in the CGRD who received the study antibiotics or reference antibiotics for more than seven consecutive days between 1 January 2014 and 31 January 2023 were eligible for inclusion. For patients with multiple hospital admissions, only the first admission was included. If a patient experienced more than one episode of receiving study or reference antibiotics for more than seven days, only the first qualifying episode was analyzed. The study antibiotics included cefazolin, flomoxef, and cefoperazone-sulbactam. Reference antibiotics consisted of other ß-lactam agents not previously reported to induce hypoprothrombinemia or hemorrhage, including cefuroxime, cefotaxime, ceftriaxone, ceftazidime, ceftazidime-avibactam, cefepime, ceftaroline, ceftolozane-tazobactam, penicillin G, oxacillin, ampicillin, ampicillin-sulbactam, amoxycillin-clavulanic acid, piperacillin, piperacillin-tazobactam, ertapenem, meropenem, doripenem, and imipenem-cilastatin. The index date was defined as the date on which the study or reference antibiotic was initiated.

Patients were excluded if they received more than one study antibiotic, both a study antibiotic and a reference antibiotic, or more than one reference antibiotic during the study period. Additional exclusion criteria included receipt of antiplatelet agents, heparin, low-molecular-weight heparin, warfarin, direct oral anticoagulants, or fibrinolytic agents (Supplementary Table S1) from one month before the index date until cessation of antibiotic therapy; abnormal PT (defined as international normalized ratio [INR] > 1.2) or computed tomography confirmed hemorrhage within three months before the index date; receipt of vitamin K within three days before or after the index date; and diagnoses of bleeding, hemorrhage, hematoma, hematuria, tarry stool, bloody stool, or melena at hospital admission.

### 2.3. Study Outcomes

The primary outcome was coagulopathy, defined as a ≥25% increase in PT from baseline. Baseline PT was defined as the closest recorded value prior to the index date; in patients without an available baseline PT measurement, coagulopathy was defined solely by an absolute PT exceeding the upper limit of normal (ULN) by more than 3 s within three days before or after cessation of antibiotic therapy [27,28,29,30]. Secondary outcomes included an INR > 1.2 and bleeding events identified using International Classification of Diseases, Ninth and Tenth Revision, Clinical Modification (ICD-9/10-CM) discharge codes (Supplementary Table S1).

### 2.4. Covariates

Recorded covariates included age, sex, Charlson Comorbidity Index (CCI), liver disease, renal function, nutritional status, duration of antibiotic therapy, and concurrent antibiotic use. The CCI was calculated using International Classification of Diseases, Ninth and Tenth Revision (ICD-9/10) codes according to the method described by Glasheen et al. [31]. Renal function was assessed using the estimated Glomerular filtration rate (eGFR). Nutritional status was determined based on nursing records during hospitalization and categorized as oral intake, nasogastric tube feeding, parenteral nutrition, or chemotherapy during the index admission. Oral intake and nasogastric tube feeding were identified from nursing documentation. Patients were classified as receiving parenteral nutrition if they were administered any parenteral nutrition formulations during the antibiotic treatment period, including Kabiven PI Emulsion, TPN SMOF Kabiven, amino acid solutions, essential amino acids, SMOFlipid 20% emulsion, fat emulsions, Lipofundin, TPN renal, TPN standard, TPN HN, or TPN hepatic. Patients were identified as undergoing chemotherapy if nursing fees related to chemotherapy administration were recorded during the index admission. Concurrent antibiotic use was defined as the administration of other systemic antibiotics, including tetracyclines, macrolides, quinolones, aminoglycosides, or glycopeptides, that were available in the CGRD and prescribed at any time between the index date and cessation of the study or reference antibiotics (Supplementary Table S2). Sensitivity analyses excluding patients without end-of-treatment PT measurements yielded results consistent with the primary analysis.

### 2.5. Statistical Analysis

Categorical variables are presented as counts and percentages, and continuous variables are expressed as means with standard deviations. Pearson’s chi-square (χ^2^) test was used to compare categorical variables, and Student’s *t* test was used for continuous variables, as appropriate. To minimize confounding due to baseline differences among treatment groups, inverse probability of treatment weighting (IPTW) was applied based on propensity scores (PS). The PS was estimated using a multivariable logistic regression model that included age, sex, Charlson Comorbidity Index, liver disease, renal function, nutritional status, duration of antibiotic therapy, and concurrent antibiotic use. Nutritional status variables (oral intake, nasogastric tube feeding, and parenteral nutrition) were highly correlated and tended to change in parallel; therefore, only oral intake was retained in the PS model to avoid multicollinearity. Each patient was weighted by the inverse probability of receiving the treatment actually administered to create a weighted pseudo-population with balanced baseline characteristics. Covariate balance before and after weighting was assessed using standardized mean differences (SMDs), with an SMD < 0.10 indicating negligible imbalance. Odds ratios (ORs) with 95% confidence intervals (CIs) were used to estimate the study antibiotics with outcomes by logistic regression models. A two-sided *p*-value < 0.05 was considered statistically significant.

## 3. Results

### 3.1. Baseline Characteristics Before Propensity Score Matching

**Figure 1:**
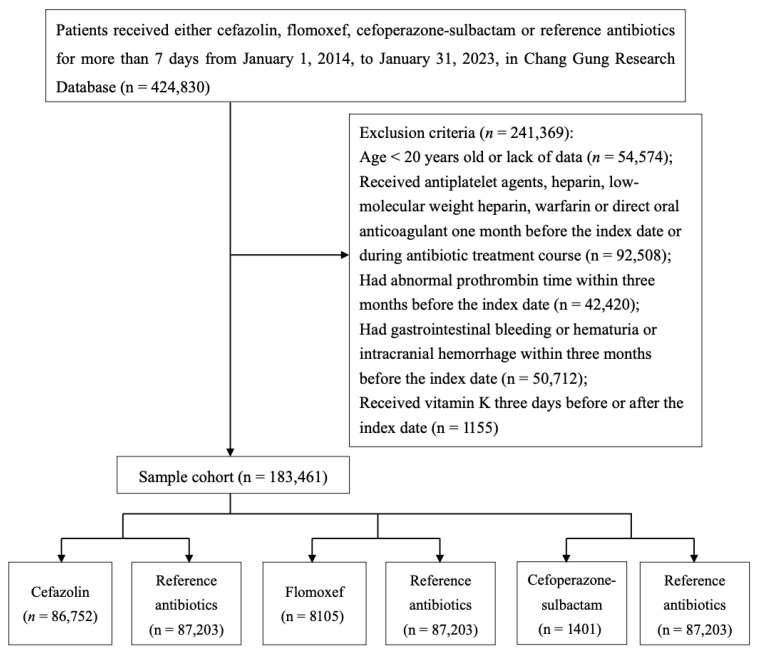
Flowchart of Patient Selection.

A total of 424,830 patients received either cefazolin, flomoxef, cefoperazone-sulbactam, or reference antibiotics. After excluding patients under 20-year-old, those with missing information of birthday, those who received antiplatelet agents, heparin, LMWH, warfarin, or direct oral anticoagulant within one month before the index date, those with coagulopathy within three months before the index date, those diagnosed with bleeding or hemorrhage within three months before the index date, and those who had received vitamin K within three days before the index date, a final cohort of 183,461 patients was included. Among these patients, 96,258 received the study antibiotics, and 87,203 received reference antibiotics (Figure 1). Significant differences were observed between the two groups in terms of age, sex, comorbidities, nutritional status, renal function, duration of antibiotics, and concurrent antibiotic use (Supplementary Table S3). Prior to IPTW adjustment, significant imbalances in baseline characteristics were observed between the study groups (cefazolin, flomoxef, and cefoperazone-sulbactam) and their respective reference cohorts (Table 1).

### 3.2. IPTW Analysis and Outcome

After applying IPTW, all baseline characteristics were well balanced across treatment groups (Table 1). Due to the comparable baseline distributions across the three treatment cohorts, stabilized weights were not utilized, and no truncation of weights was performed, as no extreme outliers were identified. The positivity assumption was confirmed through a sufficient overlap in the propensity score distributions. For the outcome analysis, weighted logistic regression models were used, with variance estimation conducted using standard error estimators.

**Figure 2:**
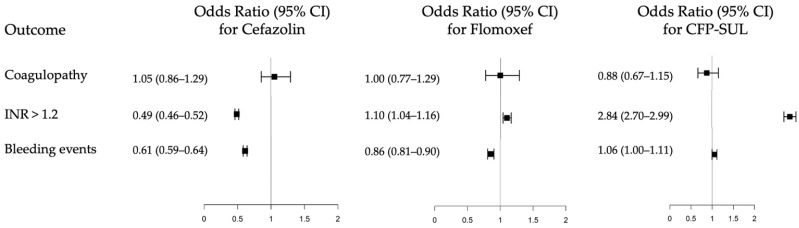
Forest plot illustrating the associations between cephalosporins of different side chains and study outcomes. Odds ratios (ORs) and 95% confidence intervals (CIs) were estimated using inverse probability of treatment weighting. Squares represent point estimates of ORs, and horizontal lines indicate 95% CIs. The vertical reference line represents an OR of 1. Abbreviations: INR, international normalized ratio; CFP-SUL, cefoperazone–sulbactam.

Compared with the reference group, patients receiving cefazolin did not have a significantly different risk of coagulopathy (odds ratio [OR], 1.05; 95% confidence interval [CI], 0.86–1.29). However, this group exhibited a lower incidence of INR > 1.2 (OR, 0.49; 95% CI, 0.46–0.52) and a lower risk of bleeding events (OR, 0.61; 95% CI, 0.59–0.64) (Table 2). Among patients receiving flomoxef, no significant association with coagulopathy was observed (OR, 1.00; 95% CI, 0.77–1.29). A modest but statistically significant increase in the incidence of INR > 1.2 was noted (OR, 1.10; 95% CI, 1.04–1.16), whereas the risk of bleeding events was significantly lower compared with the reference group (OR, 0.86; 95% CI, 0.81–0.90). For patients receiving cefoperazone–sulbactam, no significant difference in coagulopathy risk was observed (OR, 0.88; 95% CI, 0.67–1.15). Although the incidence of INR > 1.2 was markedly higher in this group (OR, 2.84; 95% CI, 2.70–2.99), this laboratory abnormality was accompanied by only a marginal increase in clinically documented bleeding events (OR, 1.06; 95% CI, 1.00–1.11). The risk estimates for the study antibiotics, relative to their respective reference groups, are illustrated in the Forest plot presented in Figure 2.

## 4. Discussion

In this retrospective cohort study, we found that the risk of coagulopathy—defined as either a ≥25% increase in PT from baseline or a PT exceeding the upper limit of normal by more than 3 s—was not significantly different among patients receiving cefazolin, flomoxef, or cefoperazone-sulbactam compared with those receiving reference antibiotics. Although the incidence of INR > 1.2 was lower in the cefazolin group, similar in the flomoxef group, and higher in the cefoperazone-sulbactam group, these laboratory abnormalities were not accompanied by corresponding increases in clinically documented bleeding events.

Cefazolin, a cephalosporin containing an MTD side chain, has been shown to interfere with hepatic microsomal vitamin K epoxide reductase activity in animal models [32]. However, clinical data regarding vitamin K status in patients receiving cefazolin are limited. In our study, cefazolin use was not associated with an increased risk of coagulopathy and was associated with lower incidences of INR prolongation and bleeding events. These findings suggest that the chemical structure of cefazolin may not be associated with clinically meaningful coagulation abnormalities or bleeding events. Given the observational nature of the study, alternative mechanisms such as bone marrow suppression, immune-mediated platelet dysfunction, or disseminated intravascular coagulation may contribute to bleeding risk in hospitalized patients receiving reference antibiotics and cannot be fully excluded [33].

Flomoxef, a cephalosporin with an HTT side chain, was not associated with an increased risk of coagulopathy or bleeding events in our analysis. While flomoxef-induced hypoprothrombinemia has been reported in experimental animal studies, clinical evidence linking flomoxef to coagulation abnormalities in humans remains limited [20,21]. A prior retrospective study by Chen et al. suggested a potential association between flomoxef use and hemorrhagic events; however, outcome ascertainment relied solely on ICD-9-CM codes [23]. In contrast, our study incorporated laboratory-based coagulation parameters, providing a more objective assessment of coagulation status. Despite this, our findings should be interpreted cautiously, and prospective studies are warranted to further clarify the coagulation safety profile of flomoxef.

Cefoperazone is reported to interfere with vitamin K metabolism through its NMTT side chain, potentially leading to prolongation of PT [19,23,28], and prophylactic vitamin K supplementation has been proposed in certain settings [29,34]. A notable finding of this study was the discordance between PT-defined coagulopathy, INR elevation, and clinically documented bleeding events. Although cefoperazone–sulbactam was associated with a higher incidence of INR > 1.2, this did not translate into increased PT-defined coagulopathy or a clear rise in clinically significant hemorrhage. Several factors may explain this pattern. Differences in monitoring intensity could have increased the detection of mild laboratory abnormalities in certain groups. In addition, bleeding events were identified using ICD discharge codes, which may underestimate minor or self-limited hemorrhagic episodes. Despite adjustment with inverse probability of treatment weighting, residual confounding and confounding by indication cannot be fully excluded. Finally, mild INR elevation does not necessarily reflect clinically meaningful impairment of hemostasis. These considerations highlight the need to interpret laboratory coagulation changes cautiously and to distinguish biochemical abnormalities from clinically significant bleeding outcomes.

Our study has several strengths. Use of the CGRD enabled access to comprehensive clinical information, including detailed diagnoses, laboratory results, medication administration records, treatment duration, and nursing documentation. In contrast to the National Health Insurance Research Database, which relies primarily on diagnostic codes, the CGRD provides granular clinical and laboratory data, allowing for more accurate identification and assessment of coagulopathy based on objective laboratory parameters.

Our study has several strengths. Use of the CGRD provided access to comprehensive clinical information, including detailed diagnoses, laboratory results, medication administration records, treatment duration, and nursing documentation. Unlike the National Health Insurance Research Database, which relies primarily on diagnostic codes, the CGRD contains granular laboratory data, allowing more precise identification of coagulopathy based on objective PT measurements. Nevertheless, several limitations warrant consideration. First, given the retrospective design, confounding by indication cannot be excluded, as antibiotic selection was determined by clinicians based on patients’ clinical condition. Validated severity scores such as SOFA or APACHE II were not available in the database, and detailed inflammatory markers or other clinical parameters reflecting acute illness severity were not systematically captured, which may have limited full adjustment for baseline disease severity. Although IPTW was applied to balance measured covariates, residual confounding remains possible. Furthermore, although the majority of covariates were well-balanced after IPTW adjustment, we observed that a covariate in the flomoxef group retained an SMD near the 0.10 threshold. This indicates a potential for a slight residual imbalance. However, since other clinical confounders strongly associated with coagulopathy were successfully balanced, it is unlikely that these minor discrepancies significantly altered the direction or magnitude of the observed odds ratios. Nonetheless, this remains a limitation inherent in weighted observational cohorts. Second, no universally accepted definition of antibiotic-associated coagulopathy exists. Although we adopted PT-based criteria used in prior studies [27,28,29,30], these definitions remain non-standard and may influence outcome classification. The observed discordance between laboratory abnormalities and clinical bleeding events further underscores the uncertainty of relying solely on biochemical parameters to assess bleeding risk. Third, nutritional status may have been misclassified despite efforts to review nursing documentation, prescriptions, and billing data. Fourth, serum vitamin K levels were unavailable because no routine clinical assay exists and such data are not captured in the CGRD. Fifth, bleeding events were identified using ICD discharge codes, which may have led to the underestimation of minor events. In addition, given the relatively low frequency of clinical bleeding outcomes, sparse data bias and imprecision in effect estimates cannot be entirely excluded. Sixth, because the study was conducted within a single healthcare system, generalizability may be limited. Finally, as an observational analysis, causality cannot be inferred. Future prospective studies incorporating standardized coagulation monitoring, direct assessment of vitamin K status, and detailed clinical evaluation of bleeding outcomes are needed to better clarify the clinical significance of antibiotic-associated coagulation abnormalities.

## 5. Conclusions

In this retrospective cohort study, no statistically significant association was observed between cefazolin or flomoxef use and clinically defined coagulopathy or bleeding events compared with reference antibiotics. Cefoperazone–sulbactam was not associated with coagulopathy under the predefined criteria but was associated with more frequent biochemical INR elevation (>1.2) and a borderline increase in bleeding risk. These findings do not indicate a consistent increase in clinically relevant coagulopathy among cephalosporins containing MTD, HTT, or NMTT side chains. However, given the observational design and the possibility of residual confounding, these results should be interpreted cautiously. Further prospective studies are needed to clarify the clinical significance of laboratory coagulation changes and the bleeding risk associated with these agents.

## Figures and Tables

**Table 1:** Baseline Characteristics of the Study Antibiotics and Reference Antibiotics.

	Cefazolin (*n* = 86,752)		Reference (*n* = 87,203)		*p* Value (Before Adjustment)	SMD (Before Adjustment)	SMD (After Adjustment)
**Age (mean ± SD)**	54.8	±16.89	59.2	±18.14	<0.001	0.254	0.003
**Male (Number, %)**	49,115	(56.6%)	44,593	(51.1%)	<0.001	0.110	0.003
**Charlson comorbidity index (number ± SD)**	1.4	±2.47	2.6	±3.49	<0.001	0.394	0.022
**Comorbidities (Number, %)**							
Liver disease	10,167	(11.7%)	12,806	(14.7%)	<0.001	0.088	0.010
**Renal function (Number, %)**					<0.001	0.463	0.064
eGFR > 60	79,810	(92.0%)	66,325	(76.1%)			
eGFR: 30–60	5140	(5.9%)	13,330	(15.3%)			
eGFR: 15–30	753	(0.9%)	3125	(3.6%)			
eGFR: <15	331	(0.4%)	1522	(1.8%)			
Renal replacement therapy	718	(0.8%)	2901	(3.3%)			
**Nutritional status** **(Number, %)**							
Oral intake	82,426	(95.0%)	72,101	(82.7%)	<0.001	0.400	0.025
NG tube feeding	2812	(3.2%)	11,798	(13.5%)	<0.001	0.378	0.053
Parenteral nutrition	1992	(2.3%)	5849	(6.7%)	<0.001	0.214	0.079
Receive chemotherapy	888	(1.0%)	2678	(3.1%)	<0.001	0.145	0.021
**Duration of antibiotics (days, %)**					<0.001	0.435	<0.001
7–10 days	70,366	(81.1%)	54,333	(62.3%)			
10–14 days	10,317	(11.9%)	18,872	(21.6%)			
14–21 days	5047	(5.8%)	11,471	(13.2%)			
>21 days	1022	(1.2%)	2527	(2.9%)			
**Concurrent antibiotic use (Number, %)**	42,143	(48.6%)	51,324	(58.9%)	<0.001	0.207	0.002
	**Flomoxef** **(*n* = 8105)**		**Reference** **(*n* = 87,203)**		***p* Value** **(Before Adjustment)**	**SMD** **(Before Adjustment)**	**SMD** **(After Adjustment)**
**Age (mean ± SD)**	57.9	±16.94	59.2	±18.14	0.82	0.077	0.006
**Male (Number, %)**	4147	(51.2%)	44,593	(51.1%)	0.96	0.001	0.019
**Charlson comorbidity index (number ± SD)**	2.3	±3.21	2.6	±3.49	<0.001	0.110	0.010
**Comorbidities (Number, %)**							
Liver disease	1698	(21.0%)	12,806	(14.7%)	<0.001	0.164	0.008
**Renal function (Number, %)**					<0.001	0.223	0.079
eGFR >60	6739	(83.2%)	66,325	(76.1%)			
eGFR: 30–60	1078	(13.3%)	13,330	(15.3%)			
eGFR: 15–30	125	(1.5%)	3125	(3.6%)			
eGFR: <15	58	(0.7%)	1522	(1.8%)			
Renal replacement therapy	105	(1.3%)	2901	(3.3%)			
**Nutritional status** **(Number, %)**							
Oral intake	6409	(79.1%)	72,101	(82.7%)	<0.001	0.092	0.026
NG tube feeding	1151	(14.2%)	11,798	(13.5%)	0.09	0.019	0.088
Parenteral nutrition	1020	(12.6%)	5849	(6.7%)	<0.001	0.200	0.100
Receive chemotherapy	116	(1.4%)	2678	(3.1%)	<0.001	0.111	0.016
**Duration of antibiotics (days, %)**					<0.001	0.102	0.067
7–10 days	5366	(66.2%)	54,333	(62.3%)			
10–14 days	1672	(20.6%)	18,872	(21.6%)			
14–21 days	929	(11.5%)	11,471	(13.2%)			
>21 days	138	(1.7%)	2527	(2.9%)			
**Concurrent antibiotic use (Number, %)**	3144	(38.8%)	51,324	(58.9%)	<0.001	0.410	0.029
	**CFP-SUL** **(*n* = 1401)**		**Reference** **(*n* = 87,203)**		***p* Value** **(Before Adjustment)**	**SMD** **(Before Adjustment)**	**SMD** **(After Adjustment)**
**Age (mean ± SD)**	66.3	±17.06	59.2	±18.14	<0.001	0.401	0.001
**Male (Number, %)**	810	(42.2%)	44,593	(51.1%)	<0.001	0.134	0.023
**Charlson comorbidity index (number ± SD)**	3.5	±3.82	2.6	±3.49	<0.001	0.228	0.015
**Comorbidities (Number, %)**							
Liver disease	145	(10.4%)	12,806	(14.7%)	<0.001	0.131	0.073
**Renal function (Number, %)**					<0.001	0.162	0.028
eGFR >60	968	(69.1%)	66,325	(76.1%)			
eGFR: 30–60	278	(19.8%)	13,330	(15.3%)			
eGFR: 15–30	77	(5.5%)	3125	(3.6%)			
eGFR: <15	34	(2.4%)	1522	(1.8%)			
Renal replacement therapy	44	(3.1%)	2901	(3.3%)			
**Nutritional status** **(Number, %)**							
Oral intake	938	(67.0%)	72,101	(82.7%)	<0.001	0.369	0.019
NG tube feeding	396	(28.3%)	11,798	(13.5%)	<0.001	0.369	0.050
Parenteral nutrition	108	(7.7%)	5849	(6.7%)	0.14	0.039	0.093
Receive chemotherapy	64	(4.6%)	2678	(3.1%)	<0.001	0.078	0.036
**Duration of antibiotics (days, %)**					<0.001	0.116	0.043
7–10 days	807	(57.6%)	54,333	(62.3%)			
10–14 days	356	(25.4%)	18,872	(21.6%)			
14–21 days	208	(14.9%)	11,471	(13.2%)			
>21 days	30	(2.1%)	2527	(2.9%)			
**Concurrent antibiotic use (Number, %)**	866	(61.8%)	51,324	(58.9%)	0.03	0.061	0.041

Abbreviations: NG tube feeding, nasogastric tube feeding; eGFR, estimated Glomerular filtration rate.

**Table 2:** Outcomes of Interest.

	Cefazolin (*n* = 86,752)		Reference (*n* = 87,203)		Weighted OR	95% CI
**Coagulopathy (Number, %)**	67	(0.08%)	109	(0.12%)	1.05	0.86–1.20
**INR > 1.2 (Number, %)**	560	(0.65%)	2160	(2.48%)	0.49	0.46–0.52
**Bleeding events (Number, %)**	1210	(1.40%)	2597	(2.98%)	0.61	0.59–0.64
Intracranial bleeding (Number, %)	699	(0.81%)	1813	(2.08%)		
Gastrointestinal bleeding (Number, %)	80	(0.09%)	378	(0.43%)		
Hematuria (Number, %)	431	(0.50%)	406	(0.47%)		
	**Flomoxef** **(*n* = 8105)**		**Reference** **(*n* = 87,203)**		**Weighted OR**	**95% CI**
**Coagulopathy (Number, %)**	10	(0.12%)	109	(0.12%)	1.00	0.77–1.29
**INR > 1.2 (Number, %)**	204	(2.52%)	2160	(2.48%)	1.10	1.04–1.16
**Bleeding events (Number, %)**	174	(2.15%)	2597	(2.98%)	0.86	0.81–0.90
Intracranial bleeding (Number, %)	114	(1.41%)	1813	(2.08%)		
Gastrointestinal bleeding (Number, %)	29	(0.36%)	378	(0.43%)		
Hematuria (Number, %)	31	(0.38%)	406	(0.47%)		
	**CFP-SUL** **(*n* = 1401)**		**Reference** **(*n* = 87,203)**		**Weighted OR**	**95% CI**
**Coagulopathy (Number, %)**	¶		109	(0.12%)	0.88	0.67–1.15
**INR > 1.2 (Number, %)**	106	(7.57%)	2160	(2.48%)	2.84	2.70–2.99
**Bleeding events (Number, %)**	48	(3.43%)	2597	(2.98%)	1.06	1.00–1.11
Intracranial bleeding (Number, %)	37	(2.6%)	1813	(2.08%)		
Gastrointestinal bleeding (Number, %)	7	(0.5%)	378	(0.43%)		
Hematuria (Number, %)	4	(0.3%)	406	(0.47%)		

¶ Masked in accordance with CGRD cell size suppression policy. Abbreviations: INR, international normalized ratio; CFP-SUL, cefoperazone–sulbactam.

## Data Availability

The data that support the findings of this study are not openly available due to confidentiality of medical records. Data are located in controlled access data storage in the Biostatistics Center, Kaohsiung Chang Gung Memorial Hospital. Data are, however, available from the authors upon reasonable request and with permission from Kaohsiung Chang Gung Memorial Hospital.

## Supplementary Materials

The following supporting information can be downloaded at: https://www.mdpi.com/article/10.3390/medicina62030519/s1. Table S1: Definition, Table S2: Antibiotics in this study, Table S3: Baseline characteristics of patients.
