# Supplementary material for: Evaluating the Risk of Coagulopathy in Cephalosporins with Different Side Chains: A Propensity Score–Weighted Study
Source: Medicina (Kaunas). 2026 Mar 11;62(3):519. doi: 10.3390/medicina62030519 (PMC13028230; doi:10.3390/medicina62030519)
Supplement: Supplementary file 1 [file medicina-62-00519-s001.zip › medicina-4030515-supplementary.pdf]

Supplementary Table S1

Definition

Antiplatelet agents, heparin, low-molecular weight heparin, warfarin, direct oral anticoagulants, or fibrinolytic agents: Warfarin, Heparin, Enoxaparin, Aspirin, Clopidogrel, Prasugrel, Ticagrelor, Clopidogrel-acetylsalicylic acid, Urokinase, Alteplase, Dabigatran, Rivaroxaban, Apixaban, Edoxaban, Fondaparinux

ICD-9 and ICD-10-CM codes of bleeding events:

Intracranial bleeding: 430, 431, 432.0, 432.1, 432.9, I60, I61, I62

Gastrointestinal bleeding: 531, 531.01, 531.2, 531.21, 531.4, 531.41, 531.6, 531.61, 532, 532.01, 532.2, 532.21, 532.4, 532.41, 532.6, 532.61, 533, 533.01, 533.2, 533.21, 533.4, 533.41, 533.6, 533.61, 534, 534.01, 534.2, 534.21, 534.4, 534.41, 534.6, 534.61, 535.01, 535.11, 535.21, 535.31, 535.41, 535.51, 535.61, 537.83, 562.02, 562.03, 562.12, 562.13, 569.85, I85.01, I85.11, K25.0, K25.2, K25.4, K25.6, K26.0, K26.2, K26.4, K26.6, K27.0, K27.2, K27.4, K27.6, K28.0, K28.2, K28.4, K28.6, K29.01, K29.21, K29.31, K29.41, K29.51, K29.61, K29.71, K29.81, K29.91, K55.21, K57.01, K57.11, K57.13, K57.21, K57.31, K57.33, K57.41, K57.51, K57.53, K57.81, K57.91, K57.93, K92.0, K92.1, K92.2

Hematuria: 599.7, N02, R31

ICD-9 and ICD-10-CM codes of covariates

Liver disease: 070.22, 070.23, 070.32, 070.33, 070.44, 070.54, 070.6, 070.9, 570, 571, 573.3, 573.4, 573.8, 573.9, V42.7, B18, K70.0, K70.1, K70.2, K70.3, K70.9, K71.3, K71.4, K71.5, K71.7, K73, K74, K76.0, K76.2, K76.3, K76.4, K76.8, K76.9, Z94.4, 456.0, 456.1, 456.2, 572.2, 572.3, 572.4, 572.8, I85.0, I86.4, K70.4, K71.1, K72.1, K72.9, K76.5, K76.6, K76.7

Supplementary Table S2

Antibiotics in this study

Hypoprothrombinemia-inducing cephalosporins: cefazolin, flomoxef, cefoperazone-sulbactam

Reference antibiotics: cefuroxime, cefotaxime, ceftriaxone, ceftazidime, ceftazidime-avibactam, ceftolozane-tazobactam, cefepime, ceftaroline, penicillin G, oxacillin, ampicillin, ampicillin-sulbactam, amoxycillin-clavulanic, piperacillin, piperacillin-tazobactam, ertapenem, meropenem, doripenem, imipenem

Concurrent antibiotics: doxycycline, tigecycline, sulbactam, trimethoprim - sulfamethoxazole, erythromycin, clarithromycin, azithromycin, clindamycin, gentamicin, amikacin, ciprofloxacin, levofloxacin, moxifloxacin, gemifloxacin, nemonoxacin, vancomycin, teicoplanin, colistin, metronidazole, linezolid, daptomycin

Supplementary Table S3.

Baseline characteristics of patients.

	Hypoprothrombinemia-inducing cephalosporins (n=96258)	Reference antibiotics (n=87203)	*p* value
Age (Mean ± SD)	55.2 ±16.97	59.2 ±18.14	< 0.001
Male (Number, %)	49115 (56.2%)	44593 (51.1%)	< 0.001
CCI (Number ± SD)	1.53 ±2.59	2.63 ±3.49	< 0.001
Comorbidities (Number, %)			
Liver disease	12010 (12.5%)	12806 (14.7%)	< 0.001
Renal function (Number, %)			< 0.001
eGFR > 60	87517 (90.9%)	66325 (76.1%)	
eGFR: 30-60	6496 (6.8%)	13330 (15.3%)	
eGFR: <15	423 (0.4%)	1522 (1.8%)	
Renal replacement therapy	867 (0.9%)	2901 (3.3%)	
Nutritional status (Number, %)			
Oral intake	89773 (93.3%)	72101 (82.7%)	< 0.001
NG tube feeding	4359 (4.5%)	11798 (13.5%)	< 0.001
Parenteral nutrition	3120 (3.2%)	5849 (6.7%)	< 0.001
Receive chemotherapy	1068 (1.1%)	2678 (3.1%)	< 0.001
Duration of antibiotics (days, %)			< 0.001
7-10 days	76539 (79.5%)	54333 (62.3%)	

10-14 days	12345 (12.8%)	18872 (21.6%)	
14-21 days	6184 (6.4%)	11471 (13.2%)	
> 21 days	1190 (1.2%)	2527 (2.9%)	
Concurrent antibiotic use (Number, %)	46153 (48.0%)	51324 (58.9%)	< 0.001

Abbreviations: CCI, Charlson comorbidity index; NG tube feeding, nasogastric tube feeding; eGFR, estimated Glomerular filtration rate
